# The Eyewitness Community Survey: An Engaging Citizen Science Tool to Capture Reliable Data while Improving Community Participants’ Environmental Health Knowledge and Attitudes

**DOI:** 10.3390/ijerph20146374

**Published:** 2023-07-16

**Authors:** Melinda Butsch Kovacic, Shereen Elshaer, Theresa A. Baker, Vincent Hill, Edith Morris, Keren Mabisi, Ian Snider, Susan Gertz, Susan Hershberger, Lisa J. Martin

**Affiliations:** 1 Cincinnati Children’s Hospital Medical Center, Department of Pediatrics, College of Medicine, University of Cincinnati, Cincinnati, OH 45229, USA; elshaese@mail.uc.edu (S.E.); theresa.baker@cchmc.org (T.A.B.); ianj.snider@gmail.com (I.S.); lisa.martin@cchmc.org (L.J.M.); 2 Department of Rehabilitation, Exercise, and Nutrition Sciences, College of Allied Health Sciences, University of Cincinnati, Cincinnati, OH 45267, USA; 3 Department of Public Health and Preventive Medicine, Faculty of Medicine, Mansoura University, Mansoura City 35516, Egypt; 4 Seven Hills Neighborhood Houses, Cincinnati, OH 45214, USA; 5 Evaluation Services Center, University of Cincinnati, Cincinnati, OH 45229, USA; morrisej@ucmail.uc.edu (E.M.); mabisikn@ucmail.uc.edu (K.M.); 6 Center for Chemistry Education, Department of Chemistry and Biochemistry, Miami University, Oxford, OH 45056, USA; gertzse@miamioh.edu (S.G.); hershbss@miamioh.edu (S.H.)

**Keywords:** reliability, environmental health, citizen science, knowledge, attitudes

## Abstract

Many youths and young adults have variable environmental health knowledge, limited understanding of their local environment’s impact on their health, and poor environmentally friendly behaviors. We sought to develop and test a tool to reliably capture data, increase environmental health knowledge, and engage youths as citizen scientists to examine and take action on their community’s challenges. The Eyewitness Community Survey (ECS) was developed through several iterations of co-design. Herein, we tested its performance. In Phase I, seven youths audited five 360° photographs. In Phase II, 27 participants works as pairs/trios and audited five locations, typically 7 days apart. Inter-rater and intra-rater reliability were determined. Changes in participants’ knowledge, attitudes, behaviors, and self-efficacy were surveyed. Feedback was obtained via focus groups. Intra-rater reliability was in the substantial/near-perfect range, with Phase II having greater consistency. Inter-rater reliability was high, with 42% and 63% of Phase I and II Kappa, respectively, in the substantial/near-perfect range. Knowledge scores improved after making observations (*p* ≤ 0.032). Participants (85%) reported the tool to be easy/very easy to use, with 70% willing to use it again. Thus, the ECS is a mutually beneficial citizen science tool that rigorously captures environmental data and provides engaging experiential learning opportunities.

## 1. Introduction

Local environments are important determinants of community health. The World Health Organization estimates that 23% of deaths and 22% of disability-adjusted life years globally can be attributed to the environment [1]. A poor environment places individuals at risk of both infectious diseases, as well as non-communicable diseases (such as stroke, heart disease, cancer, and asthma) [2]. Unfortunately, community members are often unaware of their local environment, as well as the impact the local environment can have on their own health and their community’s health.

Citizen science is a field of science where professional scientists engage amateurs from the public in research or scientific inquiry [3]. Community-based citizen science programs can improve the recognition of the local environment’s impact on health [4,5]. These programs benefit scientists, program participants, and their communities [6,7]. The benefits can include gathering local data, building local expertise, relationship-building with communities, and advocating for positive change. Citizen science observations are a unique resource that can be used by both scientists and community groups to ask and answer community-relevant questions.

Citizen science has been effectively leveraged in biodiversity monitoring, enabling the data capture of unique characteristics, which would not be possible with academic scientists alone [8]. While the use of citizen science to address issues regarding health, and specifically environmental health, has been more limited, there is the opportunity for large-scale data capture [9]. The goal would be to capture high-quality data to fill key knowledge gaps missed by traditional data gathering approaches [10]. For environmental-health-related data, a key gap is the time- and location-specific nature of environmental data. While static data are often publicly available, environmental issues, like the level of trash, are dynamic through space and time. Engaging local communities to capture such data would augment sensors and other monitoring, which may be completed by academics or public health professionals. A critical component of citizen science data is that it is high quality. A potential concern about citizen science data is the data quality and rigor [11]. Thus, it is important to appropriately train citizen scientists and to objectively review the data captured.

However, data capture should not be the only goal of citizen science, as the benefits of engagement and expertise are also important. It has long been recognized that participation in citizen science can lead to greater science knowledge gains [12] and improved awareness [13] but only when purposeful design is implemented [14]. Further, it is important to create a positive experience for citizen scientists. Projects which are frustrating due to a lack of appropriate training or complex protocols or which do not connect to a common goal can be off-putting to potential citizen scientists [15,16]. Unfortunately, similar to other experiences, once a citizen scientist has a negative experience, convincing them to participate in other projects could be difficult [17]. Thus, it is important to consider the participant perspective when developing citizen science tools. One way of developing citizen science tools which connect with the skills and interests of the potential participants is through community–academic partnerships.

Our work in community science began with a community–academic partnership that included representatives from a primarily Black under-served community. We sought to create opportunities for non-formal community-focused environmental health programming and to provide accessible, actionable data to improve environmental awareness and decision making through community and citizen science projects. Engaging citizens in data collection, we hoped, would encourage ownership of the problem, as well as greater participation in identifying and implementing solutions. As a first step, we met with community partners to identify content of interest and design the first iteration of an environmental survey through a series of meetings [18]. While participants appreciated capturing information about their community using the survey, a major concern was requiring group meetings to undertake the work. Thus, leveraging our 20+ member community–academic partnership, we modified the tool to allow completion by participants in their own time by including educational text within the tool to replace the content covered during meetings. After piloting the tool, many participants confessed that they had completely skipped the educational text we had included, which subsequently led to their failure to understand why the environmental audit was being performed [18].

The purpose of the current study was to further optimize the tool and evaluate its performance in a new group of youths, which was different from the first. Informed by our earlier attempts, we adapted the tool’s platform and developed and included more engaging educational materials to accompany the tool. Then, the tool was aptly renamed the Eyewitness Community Survey (ECS). Our research objective herein was to test the reliability of our revised ECS and to evaluate participants’ perceptions and personal benefits to ensure that we not only capture reliable data but also meaningfully engage participants and provide them with a useful experiential learning opportunity. With success, we planned to use the optimized ECS in future community-partnered environmental health research, as well as tailored educational programming within diverse communities.

## 2. Materials and Methods

### 2.1. Overall Design

The Eyewitness Community Survey (ECS) and its accompanying educational training were designed to support youths and young adult citizen scientists to make and record environmental observations within their local communities and begin to connect these observations with their community’s health. It was developed following several iterations of co-design with community members, as described previously [18]. In the current study, we evaluated the inter- and intra-rater reliability of the ECS. In Phase I, seven participants were asked to use the tool to assess five 360° photographs taken in the Cincinnati’s West End neighborhood by an academic partner (M.B.K), an intern (I.S) and a partnering West End community member. The use of photographs, versus a visit to the locations, ensured that all participants were observing the same information and was necessitated due to COVID-19 restrictions. In Phase II, 12 pairs and 1 trio of participants (n = 27) assessed at least 5 locations together within their own communities twice, typically one week apart. Both content validity and inter- and intra-rater reliability were assessed in each phase. Focus groups were held to learn more about the participants’ experiences and learnings while using the ECS in Phase II.

### 2.2. Creation of and Training to Use the ECS

Conversations with the previous users of our earlier environmental survey identified key concepts, which were important to highlight in preparing participants to use the tool: (1) defining citizen science and inviting voluntary participation in the ECS project, (2) how environment can influence human health, and (3) how to make and record observations. Informed by the prior study’s feedback [18] as well as academic and community conversations, three graphic-style stories were co-created with community members [19]. Our study team decided that the first story “Exploring the Environment” would provide an overview of the purpose of the ECS, define citizen science, and invite voluntary participation. Also, it ensured informed consent in the study. The second story “Environment is Everywhere” gave participants an educational background about the many ways that environment can impact people’s health. Topics covered included built environment, waste/recycling, and natural resources. The third and final story entitled “Becoming an Eyewitness Expert” reviewed the instructions on how to use the ECS tool. Each story used the cast of characters previously developed with the help of the We Engage for Health (WE4H) team. WE4H is a 20+ person community–academic partnership made up of researchers, educators, high school and undergraduate interns, and community members primarily from the West End—an underserved, predominantly Black neighborhood. WE4H creates and offers health promotion programming that utilizes graphic-style stories and hands-on science activities as their foundation. We have previously shown that stories are an engaging approach to sharing complex information in a low-stakes fashion [20]. Briefly, creation of each story starts with identification of a story arc. Thereafter, a written script is prepared. The scripts are shared with team members and community partners for feedback, iteratively modified, and then placed in a comic format using Comic Life software (©2023 by plasq, LLC^®^.). The graphical story drafts are then iteratively reviewed and modified by team and community members alike until a copy is finalized.

There were two phases of the study, both using the graphic-style training materials described above. Work associated with these phases was approved by the Cincinnati Children’s Hospital Medical Center Institutional Review Board. The ECS was created in REDCap [21,22]. REDCap (Research Electronic Data Capture) is a secure, web-based software platform hosted by the Center for Clinical and Translational Science and Training and designed to support data capture for research studies, providing (1) an intuitive interface to validate data capture; (2) audit trails for tracking data changes and data exports; (3) ability to seamlessly download data to common statistical packages; and (4) techniques for data integration with outside sources. In Phase I, participants attended an online meeting and read the graphic-style stories aloud together as the story characters and practiced making observations using an example photograph during the meeting. For Phase II, participants were provided embedded video links of the graphic-style stories read aloud by volunteers. The length of each video was approximately 10 min. These videos gave participants the option of asynchronously completing their training. Also, in Phase II, accompanying informational documents containing key messages shared in Videos 1 and 2 and a graphical instruction page for Video 3 could also be downloaded for review. Further, a practice module was added, which took participants approximately 3–5 min to complete. Questions that had variable interpretations in Phase I were included in this module and tested in Phase II. Briefly, participants could select photographs that they felt were the best responses to each question. If they chose responses which differed from the expected response, they were provided with the best response. In this way, we expected to help participants practice and better answer questions when they were using the tool in their own communities. All participants in Phase II were required to watch all 3 story videos and complete the practice module prior to making and recording their own observations.

For both phases, a pre-training quiz (20 questions) was given before participants read Story 2 and completed the practice module. It assessed their knowledge (10 items), attitudes (4 items), behaviors (3 items), and self-efficacy (3 items) toward environmental health. To measure changes in these parameters, participants were automatically asked to re-take the quiz after making their observations at 5 locations and then again after their 10th location. Several questions about participants’ experiences of using the ECS were also included in the post-observation quiz.

### 2.3. Observations Made with the ECS

The ECS used in both phases of this study was accessed by participants via a QR code or URL linked to a survey hosted on the REDCap platform [21,22]. The ECS was designed to help users capture community-level data within four main categories (pollution, safety, resources, and traffic and noise). The pollution category assesses litter/garbage (10 items) and air pollution and tree cover (10 items). The safety category assesses both the community’s infrastructure (23 items) and activities of people within the community (16 items). The resources category assesses public places (7 items) and the building/public services (19 items). Noise sources (2 items), noise level (2 items), and traffic density (2 items) could also be assessed. The ECS also included some general questions ascertaining the type of area observed (residential vs. commercial) and the level of building maintenance. For Phase II, participants were asked to make observations in pairs or trios but work independently. Also, they were asked to take a photograph at each location and respond to 3 additional questions, including (1) what do you see?, (2) how does this relate to your health/experiences in the community?, and (3) what changes would improve the quality of life/health? Fortunately, REDCap is able to capture geographical coordinates for each location, so these photographs and observation responses could be easily geomapped thereafter. For this reason, participants were asked to use mobile devices with an active cellular service to make their observations and to turn their location services on while participating.

In final form, the ECS consisted of a total of 117 items. While the tool was designed to be flexible and allow partnering groups to choose categories of questions to be used to answer questions that they posed, for the purpose of this validation and reproducibility study, we tested only 92 items of the 117 items. We excluded 25 items that assessed the least maintained building at each location. Given our use of 360° panoramic photographs in Phase I, we also excluded evaluation of perception-type questions, like those asking about noise, smell, and traffic density. For Phase II, for intra-rater reliability, time-sensitive questions were excluded from the analysis, and for inter-rater reliability, questions of subjective nature were excluded from the evaluation.

### 2.4. ECS and Focus Group Participants

For Phase I, we invited participants that had recently completed our virtual 6-session Citizen Science RAP (also known as Research, Ask, Promote) program [23] to take part in this study (July 2020). Those who had participated in the RAP included members of our university’s student-led Citizen Science Club, as well as family and friends of the larger WE4H team (including non-affiliated community members). We asked that participants be at least 10 years of age or older. Younger participants were asked to invite a parent/guardian to support them. Each received incentives for their participation. Phase I participants were instructed to make observations from the photographs using a laptop (most preferred) or a smartphone. They were directed to open the photograph in a new tab to switch back and forth between the photograph and the ECS observation items. Inter-rater reliability was assessed by comparing responses across participants for each photograph. To assess intra-rater reliability, participants were asked to repeat the virtual observation of at least 2 photographs approximately 7–10 days after completing the first set of observations. Feedback from participants about their experiences participating in Phase I was captured by the first author (M.B.K) via informal interviews within 4 weeks of participation.

Phase II began on 16 December 2020. The observation phase ended on 15 January 2021. A sample of middle, high school, and college students were invited to participate. Many had some prior knowledge of the WE4H program (including past or current high school or college interns). Potential participants were contacted via email to share the study information and directions. Each participant was expected to invite another participant (typically a person less familiar with WE4H programming) to work with them as a pair. Participants were instructed to make observations at 5 locations, with each location being at least one block away from the others and within their own neighborhoods across the Cincinnati Metropolitan area (Southern Ohio and Northern Kentucky). Then, participants were asked to again make observations at the same 5 locations and in the same order 1 week after completing the first set of observations. Paired observations were validated by matching each location’s geocodes (latitude and longitude) captured by REDCap. They were informed that the average anticipated time of participation was 3 to 4.5 h and that they would receive an incentive following completion of the study.

After finishing the community audit portion of the study using the ECS, Phase II participants were also invited to participate in one of three focus groups held between 18 and 29 January 2021 offered via the Zoom platform (Zoom Video Communications, San Jose, CA, USA). Those most familiar with WE4H were asked to participate in a single focus group together (N = 7). The others were divided by age, with younger participants being in one group (N = 7) and older participants in another (N = 9). Additional incentives were provided for focus group participation. Broad discovery-type questions were used, followed by specific probes as needed.

### 2.5. Statistical Analysis

Data were captured electronically via REDCap and then exported to Microsoft Excel for cleaning and processing. The data were analyzed using SPSS^®^ 26.0 (IBM, Armonk, NY, USA). Agreement for ECS individual items, ECS categories (ECS sections), and overall agreement were calculated. For Phase I, Fleiss’ Kappa was used to assess the inter-rater reliability of adult raters (more than two raters), while Cohen’s Kappa was calculated to assess inter-rater reliability for younger raters (two raters). For Phase 2, inter-rater reliability analyses were performed using Kappa statistics (Fleiss’ Kappa). For both phases intra-rater reliability analysis was performed using Cohen’s Kappa statistics. Observed % agreement was also calculated, as it provided information when one or both raters’ responses were constant. Kappa values of 0.80–1.00 were considered almost perfect agreement, 0.60–0.79 as substantial agreement, 0.40–0.59 as moderate agreement, 0.20–0.39 as fair, and 0.00–0.19 as minimal-to-slight agreement [24]. To evaluate the impact of ECS on participants, we compared the knowledge, behavior, attitudes, and self-efficacy pre-survey scores to the post-survey scores after recording the 5th and 10th observation using Wilcoxon Rank sum for paired comparisons. To graphically display some of our work, the data were imported into R v4.2.2 (R core team). For qualitative analysis, recordings were transcribed, and a codebook was developed. The data were coded and analyzed using MAXQDA (VERBI Software, MAXQDA, Berlin, Germany) by two independent qualitative researchers to identify important themes and highlight noteworthy participant comments for each.

## 3. Results

### 3.1. Study Participants

The characteristics of the study participants are described in Table 1. Overall, both phases had a similar age distribution, with most participants being white and female in both phases. Phase I participants tended to have slightly higher current educational attainment than Phase II participants. Of the Phase II participants, 23 (85.1%) shared their experiences in one of our three focus groups (having 7, 9, and 7 participants, respectively).

### 3.2. Intra-Rater Reliability

**Figure 1:**
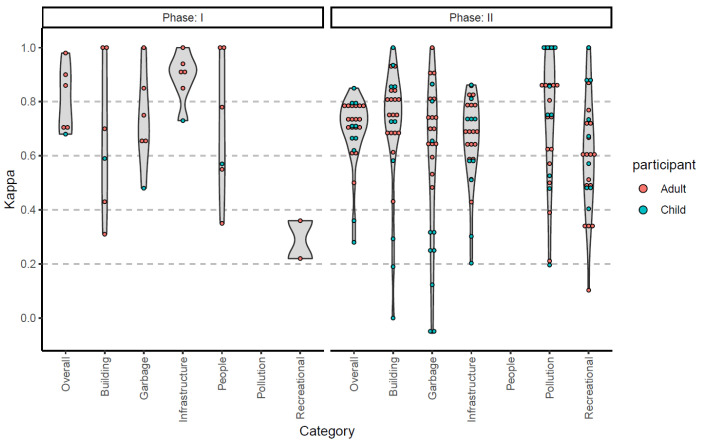
Intra-observer agreement as measured using Kappa coefficient. Phase I was performed using photographs. Phase II was performed in their local community twice, typically one week apart. Not all categories were assessed.

During Phase I, when participants evaluated photographs using the ECS, the intra-observer agreement was generally between almost perfect and substantial agreement (Figure 1, Table S1). During Phase II, participants were instructed to make observations one week apart. In reality, the difference between the two time points ranged between 6 and 21 days, with a median of 7 days. Notably, 70% of participants had an observation that fell within one day of the week of the initial observation. As a result, the intra-rater reliability was more variable (Figure 1, Table S2). While some domains exhibited less than substantial agreement (Kappa < 0.6), the overall agreement for each participating individual was substantial or high.

### 3.3. Inter-Rater Reliability

**Figure 2:**
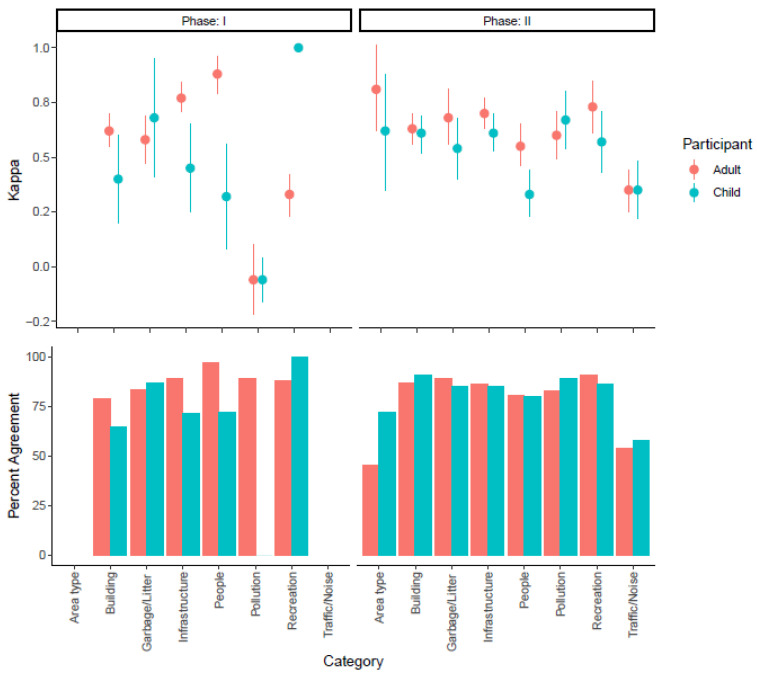
ECS inter-rater reliability (**top panel**) and agreement (**lower panel**) across the phases. Inter-rater reliability is plotted as Kappa and the 95% confidence interval. Adults are plotted in red and children in cyan. Not all categories were assessed in Phase I.

In Phase I, the participants used the tool on photographs, and 42% of the Kappa values were in the substantial-to-almost-perfect range (Figure 2, Table S3). Overall, the ECS performed well. Only 33% of the Kappa values fell in the fair-to-slight agreement range. After Phase I, informal interviews with several of the young adult participants and the caregivers of the child participants led to important insights. Participants reported having some difficulty seeing certain objects in certain photographs due to the photographic quality. Participants also shared that some questions felt too subjective and could easily be interpreted differently. They suggested creating a practice module for certain questions with these issues. In total, participants and/or study team members suggested 18 questions be modified, consistent with Phase I Kappa values.

In Phase II, a total of 65 locations from 13 neighborhoods were observed. Using the newly optimized ECS, which included educational video recordings of our graphic-style stories read aloud by characters, as well as practice modules, 63% of the Kappa values were in the substantial-to-almost-perfect range, with only 19% of the Kappa values falling in the fair agreement range (Figure 2, Table S4). Notably, the traffic/noise category had only fair agreement for both children and adult participants. This category was only evaluated in Phase II. When considering participant age, Kappa values from young adult participants tended to be higher than the Kappa values from younger youth participants. Still, the agreement demonstrated a consistency in responses for most categories.

### 3.4. Impact on Participants’ Knowledge and Attitudes

**Figure 3:**
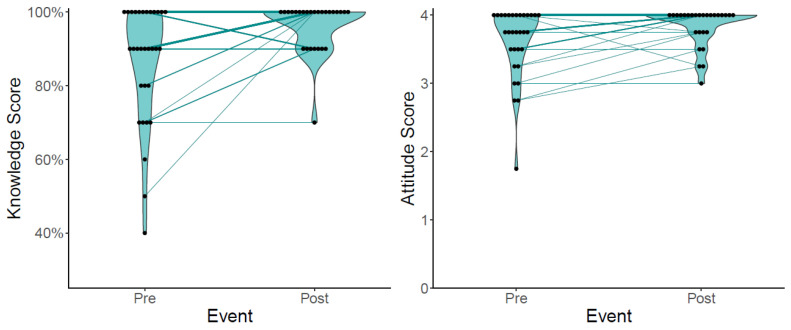
Changes in the knowledge and attitude scores pre- and post-survey (after 5 locations assessed). Individual observations are listed as points, and the distribution is provided by the violin plot. The lines show how the participants changed from pre- to post-survey, and are weighted based on the number of individuals in that category. Not all individuals who completed the pre-knowledge-score survey completed a post-knowledge-score survey. Knowledge score is calculated as the percentage of questions answered correctly. Attitude score is a weighted score based on four questions, ranging from strongly agree (4) to strongly disagree (0).

The Phase II participants took a 10-question quiz after the first “Exploring the Environment” video but before completion of the second “Environment is Everywhere” video. The post-quiz was given after participants finished making observations at 5 and 10 locations. We found that the knowledge scores significantly increased from pre- to post-survey after making observations at 5 locations (*p* = 0.005, difference 0.74 ± 1.31, Figure 3). These differences remained significant for the comparison between the pre-score and after 10 locations (*p* = 0.032, difference 0.63 ± 1.39). We also found that the attitude scores improved between the pre-score and after completing 5 locations (*p* = 0.0063, difference 0.70 ± 1.35). However, this improvement was not retained after assessing 10 locations (*p* = 0.85, −0.11 ± 1.91). Neither the behavior (*p* = 0.95 and *p* = 0.93 for 5 and 10 locations, respectively) nor the self-efficacy scores (*p* = 0.37 and 0.68 for 5 and 10 locations, respectively) on the post-surveys were significantly different than on the pre-surveys.

### 3.5. Phase II Participants’ Perceptions about the ECS

From the focus group data, five major themes emerged. Theme 1 highlighted participants’ understanding of citizen science. Indeed, nearly all focus group participants felt they had a better understanding of citizen science after participating in the study and using the ECS to make and record observations in their communities. These findings were consistent with the changes in our post-survey’s knowledge scores. As noted by one participant:

*“Another great thing about citizen science is that you don’t have to have a degree or have to come from a certain background, just be a member of the community and you have to take interest and try to improve your own health and the health of the community, gives you an opportunity just to be more engaged.”*

Another participant noted how the skills could be applied to other questions:

*“…. because you can use the same skills and methods to help your own community and yourself. Like by doing this citizen science you can like doesn’t have to be exact project we did but you could still use those skills in helping your community.”*

Theme 2 addressed learning, partnerships, and practice activities. Within this theme, there were three subthemes, including (a) learning activities helped to increase the process of conducting citizen science, (b) two-person partnerships provided a platform for discussion about the environmental survey, and (c) the ease of use of the practice activities, such as the community survey.

For example, many participants found the practice module to be helpful:

*“The video stories were fun to watch but we basically learnt the most from the practice surveys [modules] in the beginning.”*

Just as important, the inclusion of “more information buttons” within the tool itself was viewed positively:

*“In the surveys, there are a lot of like options while you’re answering the questions to click on pictures [for help in how to interpret the question] and that was really helpful to me.”*

The participants also enjoyed working with a partner:

*“The first time I did the ECS I was by myself and like by the 4th and 5th observation, I was kind of tired of it. But when I did it this time with my partner, cause we were like we were talking and you know, hanging out in between the ECS as we walked to a different locations and I didn’t feel the same fatigue with it. I actually enjoyed it.”*

Theme 3 addressed reasons for participation. Indeed, the participants had varying personal goals for participating, depending on their age and experience.

For example, some wanted to know more about their community:

*“For me, in the program kind of my personal goals were to like focus more on like looking at my community, like different parts of it, like the good and the bad of it.”*

Other participants were just looking for something to do:

*“The reason I did it was because when I started doing it, I was in quarantine from school and so I was pretty bored and so gave me a nice thing to do.”*

Theme 4 addressed participants’ perceptions of their community. Specifically, the WE4H interns shared that participation positively changed their perceptions about research and about the community and provided value.

For some, it influenced their perception of their environment:

*“I’m personally interested in medicine….., and I can see myself using those insights to think about like if someone comes into a doctor’s office, not just what information is immediately available. Like if they come in with complaints about asthma, and you are treating them, there might be more to think about in terms of where they live and what their community is like. Those insights might be useful to me.”*

For others, it opened up new career avenues:

*“I’m a first year college student so I came kind of undecided my first semester and now I’m applying to the environmental public health major. It has influenced the way that I want to pursue a career in health care, but also, knowing how the environment affects our health. I think from doing these, being a part of this project, it has helped me see how the community effects our health and that is something very important to take into account when seeing a patient or someone who goes to seek care.”*

Last, some of the participants noted changes in their perceptions about the communities they lived in. These changes were more common in the older participants:

*“I kind of have a new appreciation for planning and like urban planning for communities. Like I never knew the amount of trash cans directly affect someone’s health so if there’s not enough trash cans and people start littering, that can go in the water which can affect people’s health like I just thought it was super interesting how it all connects one way or another to one person’s health.”*

*“I definitely took notice of all the privileges that I have in my own community because I live in a place where there’s like stores and parks and all of that stuff and I know that some people don’t have that.”*

In addition to the themes that emerged, participants made specific recommendations for how the ECS should be used. These included decreasing the length and number of videos and continuing the two-person partnerships for the community survey. They affirmed that tailoring the questions to each community’s specific questions would be ideal and suggested that some questions only be asked based on prior responses (also known as using branching logic).

**Figure 4:**
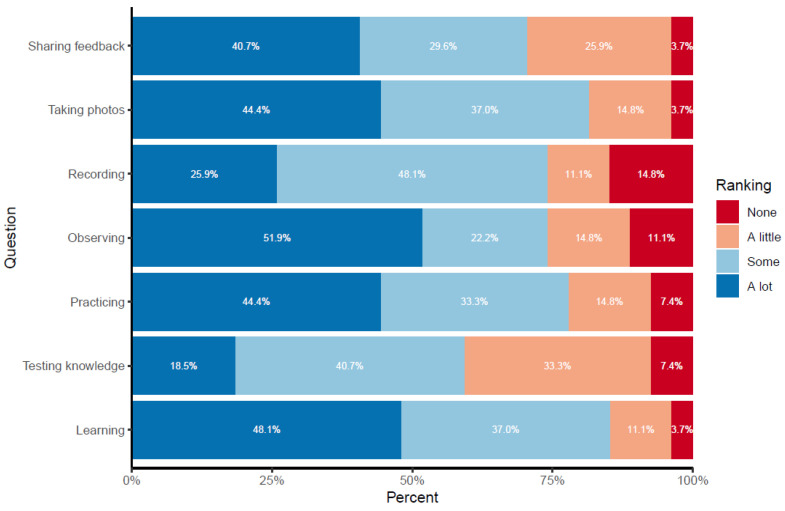
Ranking of enjoyment of the activities in the ECS after completing the first full set of observations shows overall positive perceptions.

As part of our Phase II survey, we asked for feedback on the process used in making and recording observations. These survey responses were consistent with responses from our focus group participants. Eighty-five percent of the participants noted that their participation was very easy or easy. Most participants (70%) also stated they would be willing to use the ECS again in future efforts. We also asked participants how much they enjoyed each of the activities associated with the ECS. For the most part, the great majority of participants enjoyed the activities a lot or some (Figure 4). The participants’ favorite activity was learning about citizen science and the impact of the environment on health (with 85.1% enjoying the activity a lot or some). Participants’ least favorite activity was recording the observations in the tool, with 14.8% not enjoying the activity.

A major change in the ECS between Phase I and II was the use of graphic-style story-based training videos. The survey participants’ responses indicated that the videos were very-to-somewhat useful for both how to use the tool and learning about environmental health and citizen science. Similarly, the focus group participants reported that the story videos were informative and were instrumental in creating an understanding of the project and what they were to do. However, a few focus group participants complained that the videos were redundant, particularly as many had watched them back-to-back. Moreover, the practice module was designed to repeat some of this information to ensure participants understood what was being asked of them.

## 4. Discussion

Community-based citizen science has the potential to capture important information and provide experiential learning for community participants of all ages. By creating an engaging tool, the likelihood of participants being willing to use the tool repeatedly increases. To accomplish this, we utilized graphic-style story-based educational training embedded within the ECS and completed two phases of testing. Collectively, we demonstrated that the ECS is an engaging citizen science tool that rigorously captures data and yields improved knowledge and attitudes in our participants. Specifically, the ECS was shown to have substantial-to-high intra- and inter-rater reliability, demonstrating the capacity to produce reliable data. Additionally, through pre- and post-surveys, we showed that participants’ environmental health knowledge was improved through their participation. Lastly, based on both our survey and focus group data, our results support an attitude shift not only related to environmental health but also to participating in science. As most participants had less than a college education, these results demonstrate the power of the ECS to capture high-quality data and yield substantial participant benefits.

Importantly, using a two-phase approach, we showed that most of the ECS’s data elements had Kappa values in the substantial-to-near-perfect agreement range. The demonstration of the ECS’s reliability is important, as there is a negative stigma around citizen science data [25]. Our results are consistent with prior work that has shown that such data can be of a high quality [26,27,28]. The development and design of the tool likely contributed to its reliability. Prior studies have noted that the iterative design process is a critical component of a reproducible citizen science tool [29]. The ECS was developed through multiple rounds, obtaining feedback from public and environmental health experts, stakeholders, and participants after each iteration, and modifying the tool to address concerns. We also incorporated training within the tool, as have other studies [30,31]. Indeed, our practice modules ensure the concepts being asked about were understood, as not all concepts have universal meaning. As external factors may influence reliability and to enable the assessment of reproducibility in this study, we explicitly asked participants to make and record their observations independently of other participants. In doing this, we found the youngest participants tended to have lower reliability than young adults. This is consistent with prior studies that have found that data collected by children may be of a lower quality, likely because they have less life experience, as well as a less developed skill set [32]. Nonetheless, including diverse citizen scientists is important [33]. When including children, special considerations should be utilized to maximize the data quality [34]. Ensuring they are making and recording observations within familiar environments would be helpful. For example, children from suburban regions in Phase I of our study found it challenging to classify independent row houses in our urban photographs as independent homes. Independent homes look very different in the suburbs and much more like condos. Encouraging participants to collaborate on responses while working in pairs or pairing children with adults will likely improve the data quality. Working in pairs or teams may advance participants’ social learning as well [16].

The use of the ECS resulted in higher knowledge scores, perceptions of an improved understanding of citizen science, and a greater recognition of environments and their impact on people’s health living within them. Indeed, our tool is deliberately self-reflective. For citizen science projects, the benefit to the participants is important [16]. While many citizen science projects extol participant benefits, such as knowledge gain, many projects never directly evaluate whether participants benefited or not [16]. Consistent with our findings of knowledge gain, a prior citizen science study also demonstrated increased recognition of environmental health concerns [35]. While most citizen science study participants tend to be white and have advanced degrees [36], our goal with the ECS was to develop a tool which could be used by a diverse set of participants consistent with citizen science work focused on health equity [33]. Prior studies have also noted the importance of training for successful citizen science projects [37]. We leveraged a community–academic partnership to co-create engaging educational and training materials, using graphic-style stories rather than text- or time-intensive meetings as we had with prior iterations of the ECS [18]. Graphical-style stories have been shown to be an excellent way to engage audiences and aid their understanding and retention of complex information [38]. The purposeful development of the ECS to create engaging materials and opportunities for feedback likely contributed to the improved learning outcomes [14].

We found that the ECS not only yielded reliable data and knowledge gains but also positive attitudes from the participants. As participation in community citizen science is voluntary, it is essential to create citizen science tools which are engaging and enjoyable. The ECS post-survey and focus group responses demonstrated that participants were satisfied with the processes associated with the ECS and showed short-term changes in environmental attitudes. There are several reasons why the ECS may have been perceived positively by participants. First, the ECS was developed through co-creation. Through community dialogue, we learned what local environmental concerns existed [18]. Prior work has suggested that co-creation improves accessibility and inclusivity of citizen science projects [39]. Second, the participants indicated that the videos helped them better understand the purpose of the study and what was expected of them as participants, which may have contributed to the overall positive experience. Videos were added to allow participants flexibility in when they chose to train and, if needed, re-train. We also added “cliff notes” for participants who wanted to learn information quickly and in a more traditional way. In Phase II of the collective work, the assessment was performed by pairs/trios of individuals. Working with others has been shown to increase participant satisfaction [40]. Moreover, participants noted a willingness to use the ECS in future projects. Even in citizen science projects which focus on health equity, few describe the evaluation of participant engagement [33]. The failure to appropriately engage citizen science participants may have unintended consequences, especially for youths. If participants become bored, they may become unengaged and stop or only half-heartedly collect data. This is what happened in an earlier iteration of the current tool [18]. Well-executed citizen science work can also inspire youths to consider careers in science [41]. However, citizen science projects without the appropriate engagement may achieve the opposite.

### 4.1. Limitations

Citizen scientists participating in the current study had limited racial diversity, with most identifying as White or Asian. However, the greatest environmental injustices in the United States often occurs in lower income Black and Hispanic populations [42,43]. Community–academic partnerships can help address these injustices by empowering community partners [44,45]. Importantly, the ECS was co-developed with our community partner, Seven Hills Neighborhood Houses, which serves a historically Black West End neighborhood in an under-resourced area of Cincinnati. The initial selection of topics for the ECS was based on input from West End community members [18]. Revisions of the ECS were based, in part, on feedback from the community. For example, the use of comic-book-style stories to introduce both the tool and the project were added based on recommendations from one of our community partners. As such, we anticipate that the ECS will be able to be used by a diverse set of community participants.

### 4.2. Considerations for Future Use of the ECS

Our current article focuses on making sure our tool is reliable and that we can sufficiently engage citizen scientists to reliably collect data. Completing this study makes it possible to use this tool for studies in diverse communities and for diverse purposes related to environmental health. While we saw overall positive feedback, room for improvement exists. We expect that the implementation of the tool in real-world projects will be more engaging for participants. Indeed, the focus group participants specifically mentioned that the experience would have been better if they were working toward answering a specific research question. We anticipate that having a question that participants care about, as well as answering that question, with the goal of informing a community intervention would improve motivation and interest in participation. As such, it is beneficial to partner with organizations that have research questions that using the tool can solve (improves engagement/interest of participants). Similarly, using a democratic process (what aspects to study) could also improve engagement [46]. Likewise, our Phase II participants noted there were too many observations. This was purposeful given our wish to test the reliability of as many of our tool’s questions as possible. However, we have designed the ECS to be able to easily customize the questions based on the research question(s), on the target communities to be audited, and even on the age of the participants.

To further minimize participant burden, encouraging pairs or groups of participants to survey their environments while using different tool modules would allow data capture to move more quickly. There are other optimizations of the tool that could be made as well. Other researchers have allowed citizen scientists to select the modules they want to participate in [47]. Gamification, for example, could also be used to make the tasks even more fun, particularly if there was a competitive component [48]. We could envision the next iteration of the ECS incorporating more game theory to increase engagement. Depending on the project, the inclusion of sensors to measure air pollution, temperature, or humidity, for example, could complement the data captured. Lastly, sharing information back with the community and/or the citizen scientists will be an important component of citizen science projects using the ECS. As the ECS captures the geocoordinates of the sites evaluated, it is possible to geomap the observations, create a StoryMap, and even display photos from our photovoice module at their appropriate locations. We are excited to see how this tool will be used in future environmental health citizen projects, as well as community assessments typically performed by nurses and public health professionals [49].

## 5. Conclusions

In conclusion, we created an engaging environmental audit tool for community-based citizen science projects that focuses on capturing high-quality data and has built-in training and resources on how to use the tool and helps participants learn more about their local environment and its impact on human health. Our thorough evaluation of this tool shows that it captures reliable data and enriches the citizen scientists’ knowledge and attitudes. Future work using the ECS will continue to improve upon the engagement aspect of the tool while also collecting high-quality data to study and address important local environmental health challenges.

## Figures and Tables

**Table 1:** Characteristics of the participants.

	Phase I	Phase II
N	7	27
Age (Mean ± SD)	17.3 ± 3.3	16.8 ± 3.1
Gender (n % female)	5 (71.4)	16 (59.3)
Race		
Asian	0 (0)	7 (25.9)
White	7 (100)	20 (74.1)
Ethnicity (% Latino)	2 (28.6)	3 (11.1)
Education level (n %)		
4–12th	2 (28.6)	18 (66.7)
High School	4 (57.1)	6 (22.2)
College	1 (14.3)	3 (11.1)

not mutually exclusive.

## Data Availability

The datasets generated and analyzed during the study are available from the corresponding authors on reasonable request.

## Supplementary Materials

The following supporting information can be downloaded at: https://www.mdpi.com/article/10.3390/ijerph20146374/s1, Table S1: Phase I intra-observer agreement measured using Kappa coefficient by category; Table S2: Phase II intra-observer agreement measured using Kappa coefficient by category; Table S3: Phase I inter-observer agreement measured using Kappa coefficient and percent (%) agreement; Table S4: Phase II inter-observer agreement measured using Kappa coefficient and percent (%) agreement.
