# Supplementary material for: The Eyewitness Community Survey: An Engaging Citizen Science Tool to Capture Reliable Data while Improving Community Participants’ Environmental Health Knowledge and Attitudes
Source: Int J Environ Res Public Health. 2023 Jul 16;20(14):6374. doi: 10.3390/ijerph20146374 (PMC10379192; doi:10.3390/ijerph20146374)
Supplement: Supplementary file 1 [file ijerph-20-06374-s001.zip › ijerph-2411460-supplementary.pdf]

Table S1: Phase I intra-observer agreement measured by Kappa coefficient by category

Rater	Kappa (95%CI)					
	Buildings	Infrastructure	Resources	Garbage	People	Overall
			Adults			
1	0.70 (0.51-0.88)	0.94 (0.87-1.02)	0.36 (-0.21-0.93)	0.85 (0.64-1.05)	0.78 (0.49-1.07)	0.86 (0.80-0.92)
2	0.31 (-0.07-0.69)	0.92 (0.78-1.07)	*	0.67 (0.27-1.06)	0.35 (-0.17-0.87)	0.72 (0.58-0.86)
3	1.0	1.0	*	1.0	1.0	0.98 (0.93-1.03)
4	1.0	0.85 (0.65-1.04)	*	0.64 (0.21-1.06)	1.0	0.90 (0.81-0.99)
5	0.43 (0.21-0.66)	0.90 (0.81-0.99)	0.22 (-0.06-0.51)	0.75 (0.49-1)	0.55 (0.26-0.84)	0.69 (0.61-0.77)
			Children			
6	0.59 (0.29-0.89)	0.73 (0.49-0.97)	*	0.48 (0.06-0.90)	0.57 (0.29-0.85)	0.68 (0.56-0.81)

Note: No statistics are computed for pollution because raters' observations are constants.

Table S2: Phase II intra-observer agreement measured by Kappa coefficient by category

Rater	Kappa (95%CI)					
	Garbage	Pollution	Resources	Infrastructure	Buildings	Overall
			Adults			
1	0.69 (0.38 - 1.00)	0.50 (0.14 - 0.86)	0.10 (- 0.28 - 0.49)	0.57 (0.41 - 0.74)	0.74 (0.56-0.92)	0.61 (0.50-0.72)
2	0.80 (0.55-1.06)	0.86 (0.59-1.13	0.47 (-0.13 - 1.07)	0.68 (0.50 - 0.85)	0.76 (0.58-0.94)	0.73 (0.63-0.83)
3	0.53 (0.18-0.88)	0.21 (-0.26-0.69)	0.71 (0.40-1.02)	0.66 (0.50-0.81)	0.61 (0.43-0.80)	0.61 (0.51-0.71)
4	0.65 (0.34-0.96)	0.63 (0.30-0.97)	0.73 (0.45-1.02)	0.60 (0.46-0.74)	0.93 (0.85-1.01)	0.73 (0.65-0.81)
5	0.64 (0.32-0.95)	0.57 (0.14-1)	0.51 (0.19-0.82)	0.86 (0.76-0.96)	0.67 (0.49-0.85)	0.74 (0.65-0.83)
6	0.91 (0.72-1.09)	0.86 (0.59-1.13)	0.51 (0.20-0.82)	0.81 (0.70-0.92)	0.80 (0.67-0.94)	0.79 (0.71-0.87)
7	0.65 (0.01-1.28)	0.86 (0.59-1.13)	0.87 (0.62-1.12)	0.69 (0.55-0.83)	0.82 (0.67-0.97)	0.77 (0.68-0.86)
8	0.75 (0.42-1.08)	1.0	0.62 (0.23-1.00)	0.78 (0.66-0.90)	0.74 (0.57-0.91)	0.78 (0.69-0.86)
9	0.87 (0.61-1.12)	0.87 (0.61-1.12)	0.33 (0.02-0.65)	0.84 (0.74-0.95)	0.84 (0.70-0.98)	0.80 (0.72-0.88)
10	0.91 (0.73-1.08)	0.39 (0.05-0.73)	0.59 (0.31-0.88)	0.43 (0.27-0.59)	0.43 (0.24-0.62)	0.50 (0.40-0.59)
11	0.71 (0.33-1.08)	1.0	0.61 (0.21-1.01)	0.70 (0.56-0.84)	0.69 (0.51-0.89)	0.74 (0.65-0.83)
12	0.59 (0.18-1.01)	0.86 (0.59-1.13)	0.61 (0.21-1.01)	0.68 (0.53-0.82)	0.69 (0.51-0.89)	0.71 (0.62-0.80)
13	0.48 (0.13-0.83)	1.0	0.34 (-0.02-0.69)	0.63 (0.48-0.78)	0.79 (0.66-0.93)	0.69 (0.60-0.78)
14	0.75 (0.42-1.08)	0.75 (0.45-1.05)	0.35 (0.0-0.70)	0.80 (0.67-0.93)	0.69 (0.54-0.86)	0.72 (0.63-0.81)
15	1.0	0.81 (0.50-1.11)	0.67 (0.32-1.01)	0.65 (0.49-0.81)	0.93 (0.84-1.03)	0.80 (0.72-0.88)
16	0.73 (0.45-1.1)	0.74 (0.41-1.06)	0.77 (0.52-1.02)	0.77 (0.65-0.89)	0.85 (0.72-0.98)	0.79 (0.71-0.87)
17	0.82 (0.58-1.06)	0.62 (0.26-0.97)	1.0	0.51 (0.33-0.69)	0.81 (0.64-0.97)	0.69 (0.59-0.79)
			Children			
18	0.25 (-0.20-0.70)	0.53 (0.06-0.99)	0.73 (0.45-1.02)	0.59 (0.40-0.78)	0.85 (0.69-1.01)	0.67 (0.56-0.78)
19	0.80 (0.54-1.06)	0.86 (0.59-1.12)	0.89 (0.68-1.10)	0.81 (0.69-0.94)	0.86 (0.71-1.01)	0.85 (0.68-1.02)
20	0.25 (-0.20-0.70)	1.0	0.47 (-0.13-1.07)	0.75 (0.58-0.93)	0.58 (0.34-0.83)	0.66 (0.53-0.79)
21	0.32 (-0.14-0.78)	1.0	*	0.74 (0.57-0.91)	0.94 (0.81-1.06)	0.78 (0.67-0.89)
22	0.12 (-0.12-0.36)	0.77 (0.50-1.03)	0.40 (0.20-0.60)	0.30 (0.16-0.45)	0.29 (0.06-0.53)	0.36 (0.27-0.45)
23	0.31 (0.04-0.58)	0.48 (0.19-0.77)	0.49 (0.26-0.71)	0.20 (0.05-0.35)	0.19 (0.04-0.34)	0.28 (0.19- 0.37)
24	0.87 (0.61-1.12)	0.19 (-0.30-0.69)	0.87 (0.62-1.12)	0.57 (0.35-0.80)	1.0	0.70 (0.57-0.83)
25	0.66 (0.35-0.96)	1.0	1.0	0.86 (0.73-0.99)	*	0.81 (0.71-0.91)
26	0.060 (0.13-0.02)	1.0	0.57 (0.26-0.88)	0.72 (0.58-0.86)	0.74 (0.59-0.89)	0.72 (0.63-0.81)
27	0.042 (0.10-0.01)	0.74 (0.41-1.06)	0.67 (0.34-1.01)	0.51(0.32-0.70)	0.71 (0.53-0.90)	0.62 (0.50-0.74)

Table S3: Phase I inter-observer agreement measured by Kappa coefficient and percent (%) agreement

Category	# Items	Adults (n=5)		Children (n= 2)	
		Kappa (95%CI)	% Agreement	Kappa (95%CI)	% Agreement
Area type					
Garbage/Litter	6	0.58 (0.47-0.69)	83.0	0.68 (0.41-0.95)	86.7
Pollution	3	- 0.06 (-0.22-0.10)	89.0	-0.06 (-0.16-0.04)	0.0
Recreational Sites	6	0.33 (0.23-0.42)	88.0	1.0	100
Infrastructure	14	0.77 (0.71-0.84)	89.0	0.45 (0.25-0.65	71.6
People	10	0.88 (0.79-0.96)	97.0	0.32 (0.08-0.56)	72.0
Building	9	0.62 (0.55-0.70)	79.0	0.40 (0.20-0.60)	64.6

Note: Traffic and noise constructs were not assessed in phase I as panoramic photos were assessed.

Table S4: Phase II inter-observer agreement measured by Kappa coefficient and percent (%) agreement

Category	# Items	Adults (n=9)		Children (n= 18)	
		Kappa (95%CI)	% Agreement	Kappa (95%CI)	% Agreement
Area type	1	0.81 (0.62- 1.01)	45.0	0.62 (0.35-0.88)	72.0
Garbage/Litter	6	0.68 (0.56-0.81)	88.8	0.54 (0.40-0.68)	84.7
Pollution	8	0.60 (0.49-0.71)	82.8	0.67 (0.54-0.80)	89.0
Traffic and Noise	4	0.35 (0.25-0.44)	53.6	0.35 (0.22-0.48)	58.0
Recreational Sites	6	0.73 (0.61-0.85)	90.4	0.57 (0.43-0.71)	86.0
Infrastructure	20	0.70 (0.63-0.77)	86.0	0.61 (0.53-0.70)	84.9
People	13	0.55 (0.46-0.64)	80.3	0.33 (0.23-0.44)	79.6
Building	18	0.63 (0.56-0.70)	86.7	0.61 (0.52-0.69)	90.4
